# Sphingosine-1-Phosphate Receptor 3 Induces Endothelial Barrier Loss via ADAM10-Mediated Vascular Endothelial-Cadherin Cleavage

**DOI:** 10.3390/ijms242216083

**Published:** 2023-11-08

**Authors:** Jialin Wu, Ying Liang, Panfeng Fu, Anlin Feng, Qing Lu, Hoshang J. Unwalla, David P. Marciano, Stephen M. Black, Ting Wang

**Affiliations:** 1 Center for Translational Science, Florida International University, Port Saint Lucie, FL 34987, USA; 2 Department of Environmental Health Sciences, Florida International University, Miami, FL 33199, USA; 3 Department of Immunology and Nanomedicine, Florida International University, Miami, FL 33199, USA; 4 Department of Cellular Biology and Pharmacology, Florida International University, Miami, FL 33199, USA

**Keywords:** ADAM10, ARDS, endothelial cell, S1PR3, VE-cadherin, VILI

## Abstract

Mechanical ventilation (MV) is a life-supporting strategy employed in the Intensive Care Unit (ICU). However, MV-associated mechanical stress exacerbates existing lung inflammation in ICU patients, resulting in limited improvement in mortality and a condition known as Ventilator-Induced Lung Injury (VILI). Sphingosine-1-phosphate (S1P) is a circulating bioactive lipid that maintains endothelial integrity primarily through S1P receptor 1 (S1PR1). During VILI, mechanical stress upregulates endothelial S1PR3 levels. Unlike S1PR1, S1PR3 mediates endothelial barrier disruption through Rho-dependent pathways. However, the specific impact of elevated S1PR3 on lung endothelial function, apart from Rho activation, remains poorly understood. In this study, we investigated the effects of S1PR3 in endothelial pathobiology during VILI using an S1PR3 overexpression adenovirus. S1PR3 overexpression caused cytoskeleton rearrangement, formation of paracellular gaps, and a modified endothelial response towards S1P. It resulted in a shift from S1PR1-dependent barrier enhancement to S1PR3-dependent barrier disruption. Moreover, S1PR3 overexpression induced an ADAM10-dependent cleavage of Vascular Endothelial (VE)-cadherin, which hindered endothelial barrier recovery. S1PR3-induced cleavage of VE-cadherin was at least partially regulated by S1PR3-mediated NFκB activation. Additionally, we employed an S1PR3 inhibitor TY-52156 in a murine model of VILI. TY-52156 effectively attenuated VILI-induced increases in bronchoalveolar lavage cell counts and protein concentration, suppressed the release of pro-inflammatory cytokines, and inhibited lung inflammation as assessed via a histological evaluation. These findings confirm that mechanical stress associated with VILI increases S1PR3 levels, thereby altering the pulmonary endothelial response towards S1P and impairing barrier recovery. Inhibiting S1PR3 is validated as an effective therapeutic strategy for VILI.

## 1. Introduction

Mechanical ventilation (MV) is a life-supporting strategy used in the Intensive Care Unit (ICU) to maintain adequate gas exchange in the lungs of critically ill patients [1]. However, mechanical stress associated with MV exacerbates existing lung inflammation in patients, leading to a condition known as Ventilator-Induced Lung Injury (VILI) [2]. The pathobiology of VILI resembles Acute Respiratory Distress Syndrome (ARDS) and is characterized by endothelial barrier disruption, epithelial apoptosis, leukocyte infiltration, pro-inflammatory cytokine release, and pulmonary edema [3]. In recent years, physicians have adopted a strategy to minimize tidal volume during MV for most patients, but the mortality associated with MV remains unacceptably high [2]. Furthermore, in patients presenting with more severe conditions and poor prognoses, more aggressive ventilation approaches would have to be implemented, leading to long-term lung damage [1]. Hence, there is a pressing demand to further elucidate the mechanisms behind VILI in order to develop effective therapies.

Sphingosine-1-phosphate (S1P) is a bioactive lipid metabolic product synthesized from endogenous sphingolipids, which are integral components of cellular membranes [4]. Due to their lack of intracellular S1P-degrading enzymes, platelets are the main source of S1P secretion [5]. As a result, S1P is omnipresent in the human body through the distribution of blood [6]. Indeed, researchers have characterized a plethora of physiological signaling functions mediated by S1P since the 1960s, such as cytoskeleton modulation, cell development, and lymphocyte activation [7]. However, the specific receptors of S1P were not studied until the 1990s [8]. Currently, there are five known G-protein-coupled receptors (GPCRs) for S1P, from S1P receptor 1 (S1PR1) to S1PR5. In human pulmonary endothelial cells (PECs), S1PR1 is the predominantly expressed S1P receptor that maintains barrier function and cell proliferation via its downstream Rac signaling [4]. Most research concerning S1PRs in the context of lung diseases has therefore concentrated on S1PR1. Specifically, numerous studies have exploited the S1P-S1PR1 pathway to promote vascular integrity and cell survival [9,10]. Furthermore, the most prominent drug that targets S1PRs, S1PR agonist FTY720-P, is designed to promote barrier function via its binding to S1PR1 in PECs [11].

A recent study has revealed that under mechanical stress associated with MV, S1PR3, a receptor for S1P that is expressed to a much lesser extent in PECs compared with S1PR1, is significantly upregulated [12]. S1PR3 has been shown to influence cytoskeleton organization, cell proliferation, and cell migration by coupling to Rho, indicating an opposing effect on PECs as compared with S1PR1 and its downstream Rac signaling [4]. Therefore, the cellular response to S1P could be significantly altered due to a shift in the dynamics between S1PR1 and S1PR3 expression. As MV results in significant mechanical stress, it is highly probable that the upregulation of the S1P-S1PR3 axis is responsible for compromised vascular integrity and the subsequent pathological outcomes. Thus, selectively targeting S1PR3 could be a promising strategy for containing lung damage associated with VILI. We then set out to investigate the functions and mechanisms of S1PR3 in PECs by constructing an S1PR3 overexpression model in PECs. Our study confirmed for the first time that upregulated S1PR3 remodels the cellular response towards S1P from an S1PR1-dependent barrier enhancement of S1PR3-dependent barrier disruption. Furthermore, S1PR3 overexpression induces Vascular Endothelial (VE)-cadherin cleavage and impaired endothelial adherens junction through the activities of ADAM10 and NFκB. This ADAM10-mediated VE-cadherin cleavage directly breaks the adherens junction, while the cleaved fragments further compromise cellular adherens junction by competitively binding to full-length VE-cadherin [13]. These events strongly prevent the re-dimerization of VE-cadherin, a key step to recover the disrupted barrier. We also evaluated the therapeutic potential of the S1PR3 antagonism in a murine model of VILI. To summarize, our findings indicate that the upregulation of S1PR3 during MV remodels endothelial barrier regulation, impairs VE-cadherin dynamics, and contributes to lung inflammation. Some of the results of these studies have been previously reported in the form of abstracts [14,15].

## 2. Results

### 2.1. S1PR3 Overexpression (OE) Remodels Endothelial Barrier Regulation via S1P

**Figure 1:**
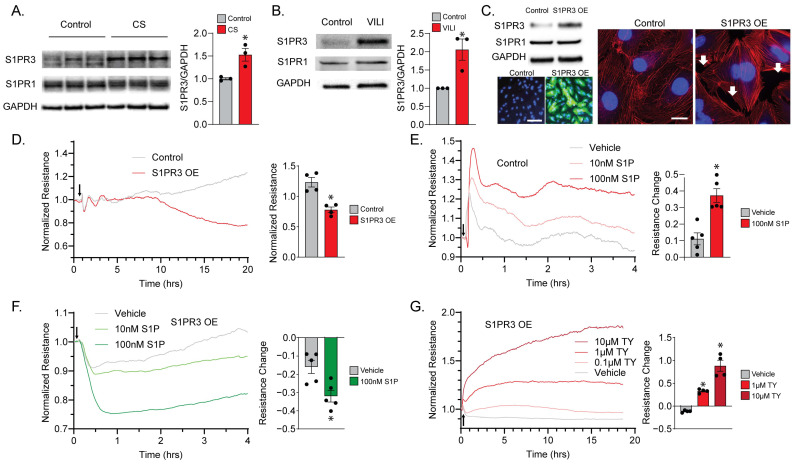
S1PR3 overexpression (OE) remodels endothelial barrier function. (**A**) S1PR3 and S1PR1 protein levels in control cells and PECs exposed to cyclic stretch (CS, 8 h) were analyzed via Western blot (*n* = 3, * *p* < 0.05). (**B**) S1PR3 and S1PR1 levels in lung tissues from control mice and mechanically ventilated mice were analyzed via Western blot. (*n* = 3, * *p* < 0.05). (**C**) S1PR3 and S1PR1 levels in control cells and S1PR3 OE cells and S1PR3 overexpression was also confirmed by co-expressed GFP (green) (scale bar = 50 μm). Immunofluorescence imaging of DAPI (blue) and F-actin (red) was also performed (scale bar = 20 μm). Paracellular gaps (white arrowheads) were found in S1PR3 OE cells. (**D**) Normalized TER measurements were plotted against time for control cells and S1PR3 OE cells. The time point at which adenovirus was added is marked with a black arrow. Normalized resistance values were compared at 20 h between the two groups (*n* = 4, * *p* < 0.05). (**E**) Normalized TER measurements with S1P challenge were plotted against time in control endothelial cells. These control cells were challenged with vehicle only (culture media) or S1P (10 and 100 nM). The time point of vehicle/S1P addition is marked with a black arrow. Normalized resistance values were compared at 2 h between the two groups (*n* = 5, * *p* < 0.05). (**F**) Normalized TER measurements with S1P challenge were plotted against time in S1PR3 OE endothelial cells. These S1PR3 OE cells were challenged with vehicle only (culture media) or S1P (10 and 100 nM). The time point of vehicle/S1P addition is marked with a black arrow. Normalized resistance values were compared at 2 h between the two groups (*n* = 5, * *p* < 0.05). (**G**) Normalized TER measurements with S1P challenge were plotted against time in S1PR3 OE endothelial cells with vehicle or TY-52156 (0.1, 1, and 10 μM) pre-treatment for 24 h. The time point of S1P addition is marked with a black arrow. Normalized resistance values were compared at 18 h in three groups (*n* = 4, * *p* < 0.05 compared with control group).

We first confirmed the upregulation of S1PR3 upon ventilation or MV-associated mechanical stress. S1PR3 protein levels were significantly elevated in PECs exposed to cyclic stretch (CS, 8 h, Figure 1A), mimicking VILI-associated mechanical stress. Notably, there were no significant changes in S1PR1 protein levels. In addition, in the lung tissues of a murine model of VILI (40 mL/kg, 4 h), S1PR3 protein was also significantly upregulated, while S1PR1 protein levels remained unchanged (Figure 1B).Therefore, we proceeded to establish an S1PR3 overexpression (OE) PEC model to study the specific downstream effects of S1PR3. After being exposed to Ad-S1PR3 or Ad-Null (Vector Biolabs) for 24 h, S1PR3 expression was notably elevated in S1PR3 OE PECs with no discernible impact on S1PR1 levels. Positive transduction and overexpression were further confirmed by co-expressed GFP in transduced cells. In addition, S1PR3 OE induced actin stress fiber formation and paracellular gaps in PECs, consistent with the effects of Rho, a transcription factor downstream of S1PR3 [16,17] (Figure 1C).

After treating a single layer of PECs plated on ECIS plates with Ad-S1PR3, the resistance of S1PR3 OE cells could be differentiated from the control cells (treated with Ad-Null) approximately 8 h after treatment and continued to decrease (Figure 1D), suggesting S1PR3-dependent PEC barrier disruption. To further examine the cellular response towards S1P, we treated both the control cells (Figure 1E) and the S1PR3 OE cells (Figure 1F) with 10 nM and 100 nM S1P. We were able to observe an S1P dose-dependent acute increase in the resistance of control cells, which is consistent with the response of lung endothelial cells in other studies [18]. Importantly, since S1P binds to its receptors while coupling to high-density lipoprotein (HDL), using serum-free medium would prevent us from observing the acute effect of S1P on cell permeability [19]. Therefore, we used regular medium with FBS as our vehicle to deliver S1P treatment, which contained an estimated concentration of 4 nM S1P (5% FBS) [20]. Henceforth, vehicle treatment in control cells mimicked a lower dose of S1P treatment compared with our 10 nM and 100 nM S1P groups (Figure 1E). In contrast, S1PR3 OE cells exhibited an acute decrease in cellular resistance upon vehicle or S1P treatment, which was also dose-dependent (Figure 1F). Furthermore, the S1PR3 antagonist TY-52156 (TY) was able to rescue the barrier-enhancing cellular response towards S1P in S1PR3 OE cells dose-dependently (1 μM and 10 μM) (Figure 1G). These data demonstrate that S1PR3 overexpression, which can be induced by VILI-associated mechanical stress, remodels the endothelial barrier response towards S1P.

### 2.2. S1PR3 OE Induces VE-Cadherin Cleavage in PECs

**Figure 2:**
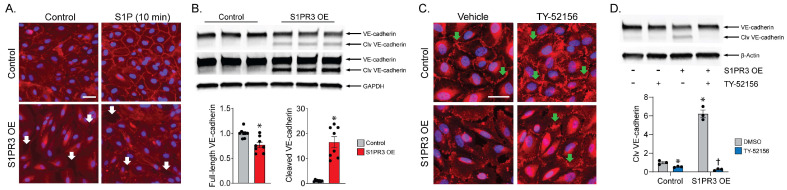
S1PR3 OE disrupts VE-cadherin redistribution and causes VE-cadherin cleavage in endothelial cells. (**A**) Immunofluorescence imaging of VE-cadherin (red) and DAPI (blue) in control and S1PR3 OE cells treated with vehicle/S1P for 10 min. Paracellular gaps are marked with white arrows. Scale bar = 50 μm. (**B**) VE-cadherin levels in control. S1PR3 OE cells were analyzed via Western blot. Both full-length VE-cadherin and cleaved (clv) VE-cadherin from the two groups were compared (*n* = 8, * *p* < 0.05). Signal intensity was normalized to GAPDH. Two images captured using a higher and lower exposure time are shown. (**C**) Immunofluorescence imaging of VE-cadherin (red) and DAPI (blue) in control and S1PR3 OE cells pre-treated with vehicle or TY-52156 (10 μM) for 24 h. S1P was then administered for 5 h. VE-cadherin junctions are marked with green arrows. Scale bar = 50 μm. (**D**) VE-cadherin levels were analyzed via Western blot in control and S1PR3 OE cells treated with vehicle or TY-52156 (10 μM) for 24 h. Cleaved (Clv) VE-cadherin from the four groups was compared (*n* = 3, * *p* < 0.05 compared with control/DMSO group, † *p* < 0.05 compared with S1PR3 OE/DMSO group).

We examined cell adherens junctions by staining VE-cadherin to confirm the effects of S1PR3 on PEC barrier regulation. Compared with control cells, VE-cadherin in S1PR3 OE cells failed to localize to the periphery of the cells after S1P treatment (Figure 2A). Therefore, we performed an immunoblot of VE-cadherin and found that the VE-cadherin (~130 kD) protein level was reduced in S1PR3 OE cells, while a cleaved VE-cadherin band (~100 kD) emerged below the main band in S1PR3 OE cell samples (Figure 2B). This suggests S1PR3 OE induced VE-cadherin cleavage, which might have in turn impaired the recovery of the PEC barrier. Furthermore, we were also able to see that the S1PR3 inhibitor TY (10 μM) restored peripheral VE-cadherin and eliminated paracellular gaps in S1PR3 OE cells using immunofluorescence (Figure 2C). We also confirmed VE-cadherin cleavage was dependent on S1PR3 activity, with TY treatment (10 μM) completely amending the cleavage, as shown by immunoblotting (Figure 2D). These data demonstrate that S1PR3 OE mediated the disruption of adherens junction, an important component of the endothelial barrier, via VE-cadherin cleavage.

### 2.3. ADAM10 Mediates VE-Cadherin Cleavage

**Figure 3:**
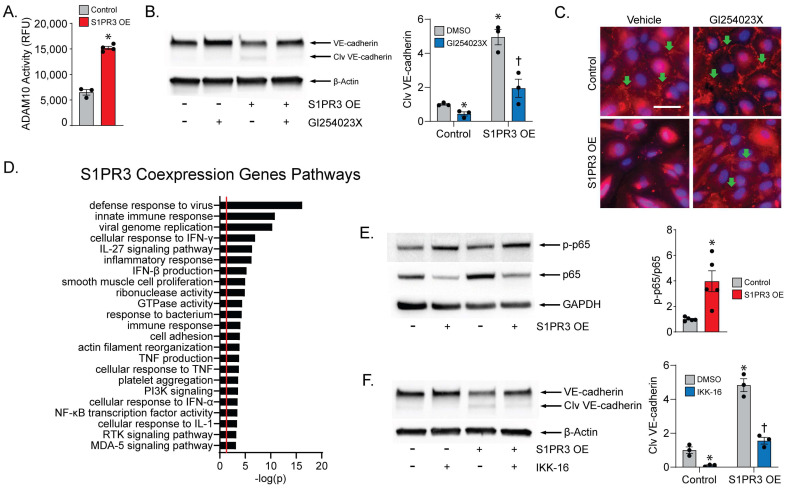
S1PR3 OE-induced VE-cadherin cleavage is ADAM10- and p65 activity-dependent. (**A**) The enzymatic activity of ADAM10 was assayed in both control and S1PR3 OE cells (* *p* < 0.05). (**B**) VE-cadherin levels were evaluated via Western blot in both control and S1PR3 OE cells treated with vehicle or GI254023X (20 μM) for 24 h. Cleaved VE-cadherin was analyzed in all four groups (*n* = 3, * *p* < 0.05 compared with control/DMSO group, † *p* < 0.05 compared with S1PR3 OE/DMSO group). (**C**) Immunofluorescence imaging of VE-cadherin (red) and DAPI (blue) in control and S1PR3 OE cells treated with vehicle or GI254023X (20 μM) for 24 h. S1P was then administered for 5 h. VE-cadherin junctions are marked with green arrows. Scale bar = 50 μm. (**D**) Enriched biological process pathways (Gene Ontology) were analyzed for S1PR3 co-expressed genes. The red line is used to mark *p* < 0.05. (**E**) Phosphorylated and non-phosphorylated NFkB (p65) was examined via Western blot in both control and S1PR3 OE cells. The ratio of p-p65/p65 was then calculated (*n* = 5, * *p* < 0.05). (**F**) VE-cadherin was examined in both control and S1PR3 OE cells treated with vehicle or IKK-16 (1 μM) for 24 h. Cleaved VE-cadherin was analyzed in all four groups (*n* = 3, * *p* < 0.05 compared with control/DMSO group, † *p* < 0.05 compared with S1PR3 OE/DMSO group).

Next, we aimed to characterize the specific mechanism of VE-cadherin cleavage in S1PR3 OE PECs. ADAM10, a highly expressed metalloprotease in PECs, was reported to cleave VE-cadherin in a similar fashion to what we observed, while the ADAM10 inhibitor GI254023X (GI) could attenuate induced barrier function loss [13]. Therefore, we measured the enzymatic activity of ADAM10 and saw an increase in S1PR3 OE cells compared with control cells (Figure 3A). To confirm the specificity of this finding, we treated the cells with GI (20 μM) and found VE-cadherin cleavage to be significantly reduced in S1PR3 OE cells (Figure 3B). Furthermore, we found that the recovery of VE-cadherin junctions in S1PR3 OE cells after GI treatment was similar but weaker compared with that after TY treatment (Figure 3C).

In the literature, it was demonstrated that NFκB, a crucial regulator of cellular inflammatory pathways, is associated with the activation of ADAM10 [21]. Using an S1PR3 overexpression dataset in NCBI (NIH GEO # GSE149238), we conducted a pathway analysis for genes upregulated along with S1PR3 and found a plethora of pathways associated with inflammation, including NFκB activity, that were significantly enriched in these genes (Figure 3D). In our system, we also found elevated NFκB phosphorylation (p-p65) levels in S1PR3 OE cells (Figure 3E), and p65 translocation to the nucleus (Supplemental Figure S1). In addition, p65 was found to be degraded as an outcome of DNA binding and activation [22] (Figure 3E). To confirm that NFκB activation specifically triggered the cleavage of VE-cadherin, we employed IKK-16, an IkB Kinase (IKK) inhibitor that inhibits the translocation of NFκB to the nucleus, and observed ameliorated VE-cadherin cleavage in S1PR3 OE cells after IKK-16 treatment (1 μM) (Figure 3F). These data confirm that ADAM10-dependent VE-cadherin cleavage is mediated by S1PR3 via activated NFκB.

### 2.4. S1PR3 Inhibitor Prevents Experimental VILI

**Figure 4:**
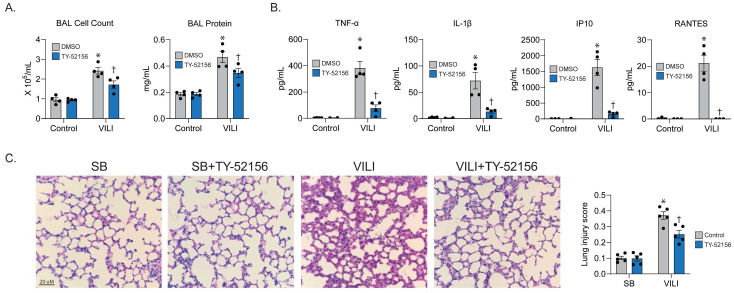
S1PR3 inhibitor TY52156 attenuates VILI in vivo. C57/B6 male mice (8 weeks) were anesthetized, treated with DMSO or TY-52156 (10 mg/kg, 30 min before ventilation), and left to breathe without assistance, a mode known as Spontaneous Breathing (SB), or placed on mechanical ventilation (4 h, 40 mL/kg). Bronchoalveolar lavage (BAL) fluid was then extracted for the analysis of (**A**) total cell count, protein concentration, and (**B**) pro-inflammatory cytokine levels (*n* = 4, * *p* < 0.05 compared with SB/DMSO group, † *p* < 0.05 compared with VILI/DMSO group). (**C**) Lung tissues were harvested and HE-stained, and the lung injury score was then evaluated (*n* = 5, * *p* < 0.05 compared with SB/DMSO group, † *p* < 0.05 compared with VILI/DMSO group).

To confirm our in vitro findings, we conducted mechanical ventilation in mice and examined the effect of blocking S1PR3 using TY (10 mg/kg) on VILI-associated lung damage. In bronchoalveolar lavage (BAL) fluid extracted from mice, we found an attenuated cell count and protein concentration for ventilated mice that received TY pre-treatment (Figure 4A). On the other hand, the levels of multiple cytokines (TNFα, IL1β, IP10, and RANTES) related to pro-inflammatory pathways were also significantly amended for TY-treated VILI mice (Figure 4B). Furthermore, in the lung histology slides from TY-treated VILI mice, we observed a dissipation of neutrophil infiltration and quantified a significant reduction in the lung injury score using ATS criteria (Figure 4C), thereby confirming the therapeutic effect of TY in vivo.

## 3. Discussion

Ventilator-induced lung injury (VILI) is a serious and unpredictable syndrome that can have devastating consequences in the ICU, despite being more manageable than pre-ICU critical illnesses such as sepsis-induced ARDS. A mechanistic understanding of the underlying pathophysiology, which is characterized by prolonged inflammation and impaired endothelial barrier recovery, is crucial for the development of effective therapies to improve ICU survival. In this study, we found that mechanical stress-induced S1PR3 remodels the regulation of PEC barrier function by endogenous S1P, instigates the cleavage of VE-cadherin via activated ADAM10, and retards barrier function recovery through initiating a cytokine storm. We also confirmed the efficacy of S1PR3 inhibition as a therapeutic approach in a pre-clinical model of VILI. These findings strongly suggest that S1PR3 plays a significant role in PEC injury during VILI and is a viable therapeutic target for this condition.

### 3.1. S1P and S1P Receptors in Acute Lung Injury

Endogenous S1P is a highly bioactive lipid with a circulating concentration of 0.1–1.2 μM and a high affinity for its receptors (EC50 ~1 nM) [23]. Due to the fact that a small level of S1P is present in animal serum, our vehicle treatment (culture media with 5% FBS) also exhibited S1P-driven effects on PEC barrier function even without exogenous S1P (Figure 1E,F). S1PR1 and S1PR3 are the two main S1P receptors, playing crucial and opposite roles in the regulation of endothelial barrier function in the lung. Activation of S1PR1 by S1P promotes the assembly of cortical actin filaments and strengthens adherens junctions, leading to improved barrier integrity. In contrast, S1PR3 activation by S1P leads to endothelial barrier dysfunction via small GTPase RhoA activation and stress fiber formation. The role of S1PR3 in endothelial injury during ARDS and VILI is poorly studied compared with that of S1PR1, since lung endothelium usually exhibits an S1PR1-dominant phenotype. For the first time, we have demonstrated with our study that S1PR3 OE, which mimics VILI-induced S1PR3 upregulation, enables S1PR3 to outcompete the predominant receptor S1PR1 and remodels PECs to exhibit an S1PR3-dominant phenotype in regard to barrier regulation by endogenous S1P. In addition, S1PR3 is upregulated in lung microvascular endothelium during ALI [12] and PECs during VILI (Figure 1A). S1PR3 might also be upregulated in other types of lung cells during ALI [24], suggesting a boarder but more impactful role of S1PR3 in ALI development.

### 3.2. S1PR3 and Inflammation

Prior to this study, S1PR3 had already been shown to play an important role in innate immune responses, in which the loss of S1PR3 causes a significant decrease in the bacteria-killing capacity of macrophages [25]. Considering that S1PR3 is also upregulated by LPS challenge in macrophages [26], we can conclude that S1PR3 served an important function in innate immunity during our evolutionary history. Nevertheless, with the development of anti-septic and anti-viral treatments, exacerbated and uncontrolled inflammation combined with MV became an often more significant threat to the life of ICU patients than pathogens. The inhibition of inflammatory responses is therefore beneficial for the survival of patients on MV. Here, we described the potential role of S1PR3, a GPCR upstream of the well-established endothelial inflammatory hub NFκB, as an inflammatory switch. Henceforth, the inhibition of S1PR3 and its downstream pathways serves the dual purpose of preserving vascular integrity and amending excessive inflammation. Furthermore, increased S1PR3 elicited a cell remodeling process similar to EMT that made PECs more migratory and fibrotic [27], which suggests that inhibiting S1PR3 could potentially prevent scarring, fibrosis, and long-term tissue damage in patients, thereby providing lasting medical benefits.

### 3.3. Therapeutic Potential of TY-52156

To date, there have been more than 600 clinical trials targeting Rho signaling [28,29,30]. In contrast, no clinical trial targeting S1PR3 has ever been conducted. It could thus be beneficial to target S1PR3 in a clinical trial, as S1PR3 is not only an upstream regulator of Rho but also a mediator of other pathways, like VE-cadherin cleavage and NFκB-mediated inflammation. In addition, most existing S1P-modulated drugs targeting S1PR1 agonistically have the side effect of inducing downregulation of S1PR1 [31]. Therefore, this approach leads to less-than-optimal clinical effects and long-term pulmonary function decompensation due to immunosuppression. In contrast, inhibiting S1PR3, a negative regulator of vascular barrier function upregulated during mechanical stress, prevents modulating S1PR1 expression, therefore ensuring a specific therapeutic effect during VILI with few side effects under normal lung conditions. However, S1PR3 is also expressed in the cardiovascular system, kidney, and spleen [32,33]. The inhibition of S1PR3 could also lead to hypertension, bradycardia, macular edema, reduced pulmonary function, hepatic adverse effects, and neoplasm [34]. Inhalation delivery of S1PR3 inhibitors via aerosol or nanoparticles, which facilitates the direct delivery of drugs deeply into the airways, will mitigate these effects. Meanwhile, as a major limitation, TY-52156, although a selective inhibitor of S1PR3, may carry notable off-target effects, including inhibiting other S1P receptors with relatively lower affinity [35]. A newer and more specific S1PR3 antagonist is needed for the next phase of VILI drug development.

## 4. Materials and Methods

### 4.1. Cell Culture and Reagents

PECs were purchased from Lonza Bioscience (Morristown, NJ, USA) (LOT# 657513) and grown in human endothelial cell culture media from Lifeline Cell Technology. PECs between passages 6 and 8 were used in all experiments. An adenovirus expressing human S1PR3 (Ad-S1PR3) was purchased from Vector Biolabs to overexpress S1PR3 for 24 h in PEC (S1PR3 OE). Ad-GFP or Ad-Null (Vector Biolabs, Malvern, PA, USA) was used as the control. S1P, S1PR3 antagonist TY-52156 (Sigma-Aldrich, St. Louis, MO, USA), ADAM10 inhibitor GI254023X (Sigma-Aldrich, St. Louis, MO, USA), and bovine serum albumin were obtained from Sigma-Aldrich. IKK-16 (Tocris Bioscience, Bristol, UK) was purchased from Tocris.

### 4.2. Transendothelial Electrical Resistance (TER)

PECs were seeded and cultured in an 8-well culture array (8W10E+ from Applied Biophysics, Troy, NY, USA) until reaching confluency. Transendothelial Electric Resistance (TER) was then continuously measured every 20 s using the Electric Cell-substrate Impedance Sensing (ECIS) system (Applied Biophysics) at 4000 Hz. The resulting TER values were summarized for statistical analysis.

### 4.3. Western Blotting

After treatment, PECs were washed with cold phosphate-buffered saline (PBS), scraped down, and homogenized in RIPA lysis buffer with a protease inhibitor cocktail and phosphatase inhibitor cocktail (Sigma-Aldrich, St. Louis, MO, USA). Lysates were centrifuged at 13,000× *g* for 15 min at 4 °C and the protein extract supernatant was collected. After being boiled with Laemmli Sample Buffer (Bio-Rad, Hercules, CA, USA) and β -Mercaptoethanol, extracted protein (10 μg/lane) was separated using 4–20% gradient SDS PAGE gels (Thermo Scientific, Rockford, IL, USA) and transferred to PVDF membranes. The membrane was then blocked with blocking buffer (5% BSA in TBST) for 1 h and incubated with corresponding antibodies in blocking buffer overnight at 4 °C. The membrane was then washed in TBST and incubated with appropriate secondary antibodies conjugated to HRP for 1 h at room temperature. After further washing in TBST, bands were visualized via chemiluminescence (West-Femto, Pierce, Rockford, IL, USA) and quantified using an iBright Imaging System (Thermo-Fisher Scientific, Waltham, MA, USA). S1PR1 (PA11040) and VE-cadherin (361900) antibodies are from Thermo-fisher (Waltham, MA, USA), β -actin (8457S), p-p65 (3033S), and p65 (8242S) antibodies are from cell signaling (Danvers, MA, USA), and GAPDH (G8795) antibody is from Sigma-Aldrich, while S1PR3 antibody (A1404) is from Abclonal (Woburn, MA, USA).

### 4.4. Immunofluorescence

Control and S1PR3 overexpression (S1PR3 OE) cells were grown on glass coverslips placed in 24-well plates. After the cells were confluent, 4% formaldehyde (in PBS) was used to fix the cells overnight. After washing with PBS, the cells were then incubated for 20 min in 0.5% TritonX-100 (*v*/*v*) in PBS or −20 °C methanol for permeabilization. For F-actin staining, cells were washed with room temperature (RT) PBS and incubated with Alexa Fluor 488 Phalloidin (Thermo-fisher) in PBS for 20 min. For VE-cadherin staining, cells were washed with RT PBS, incubated with blocking buffer (5% bovine serum albumin in PBS) for 1 h, and incubated with VE-cadherin antibody (ab33168) in blocking buffer overnight. On the next day, appropriate secondary antibodies in blocking buffer were incubated with the coverslips for 1 h at room temperature. The nucleus was then stained with DAPI for 20 min. These coverslips were examined, and representative images were captured using a Keyence BZ-X810 microscope (Keyence, Itasca, IL, USA).

### 4.5. Biological Pathway Analysis

A dataset within the GEO Datasets on NCBI, GSE149238, was used to identify co-expression genes of S1PR3, genes that are significantly upregulated along with S1PR3 overexpression. The biological process pathways (Gene Ontology) associated with these co-expressed genes were then characterized using NIH DAVID (https://david.ncifcrf.gov/ accessed on 1 February 2023) and summarized along with their −log (p).

### 4.6. Mouse VILI Model

C57/B6 mice (8 weeks male) received TY-52156 (10 mg/kg) or vehicle DMSO (I.P.) 30 min before mechanical ventilation (40 mL/kg, 4 h). Bronchial alveolar lavage (BAL) fluid was extracted. Then, the cell and protein contents of BAL were analyzed. BAL cytokines were measured with Luminex 200 (Bio-techne, Minneapolis, MN, USA). Lung injury score from histology analysis was assessed using ATS criteria [36].

### 4.7. Statistical Analysis

GraphPad Prism 9.5.0 was used to conduct statistical analysis. Mean ± SEM was calculated in all experiments, while statistical significance was determined using a *t*-test or an ANOVA test with multiple comparisons. *p* < 0.05 was considered statistically significant.

## 5. Conclusions

Mechanical stress associated with VILI upregulates S1PR3, leading to the reprogramming of endothelial barrier function and inflammation. Specifically, S1PR3 mediates the cleavage of VE-cadherin by ADAM10, thereby impeding the recovery of broken endothelial adherens junctions. Furthermore, S1PR3 overexpression activates NFκB-dependent inflammatory signaling cascades. Targeting S1PR3 yields ameliorated VILI-associated damage using both in vitro and in vivo models. Therefore, follow-up studies should be conducted to address the pharmacological development of a clinically viable S1PR3 inhibitor.

## Data Availability

All original research data supporting reported results can be made available upon request.

## Supplementary Materials

The following supporting information can be downloaded at: https://www.mdpi.com/article/10.3390/ijms242216083/s1.
