# Supplementary material for: Sphingosine-1-Phosphate Receptor 3 Induces Endothelial Barrier Loss via ADAM10-Mediated Vascular Endothelial-Cadherin Cleavage
Source: Int J Mol Sci. 2023 Nov 8;24(22):16083. doi: 10.3390/ijms242216083 (PMC10671260; doi:10.3390/ijms242216083)
Supplement: Supplementary file 1 [file ijms-24-16083-s001.zip › ijms-2662268-supplementary.pdf]

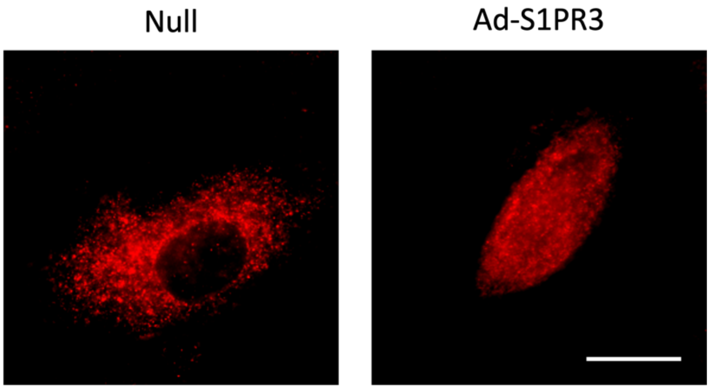

Supplementary Figure S1. Immunofluorescence images of p65 (red) in control (Ad-NULL) and S1PR3 OE cells. p65 translocate to the nucleus was observed in S1PR3 OE cells. （scale bar=20 μm）
